# The Use of Artificial Neural Networks to Predict the Physicochemical Characteristics of Water Quality in Three District Municipalities, Eastern Cape Province, South Africa

**DOI:** 10.3390/ijerph18105248

**Published:** 2021-05-14

**Authors:** Koketso J. Setshedi, Nhamo Mutingwende, Nosiphiwe P. Ngqwala

**Affiliations:** Environmental Health and Biotechnology Research Group, Division of Pharmaceutical Chemistry, Faculty of Pharmacy, Rhodes University, Makhanda 6139, South Africa; kj.setshedi22@gmail.com (K.J.S.); nmutingwende@gmail.com (N.M.)

**Keywords:** artificial neural network, artificial intelligence, physicochemical, prediction, multilayer perceptron, radial basis function, water quality

## Abstract

Reliable prediction of water quality changes is a prerequisite for early water pollution control and is vital in environmental monitoring, ecosystem sustainability, and human health. This study uses Artificial Neural Network (ANN) technique to develop the best model fits to predict water quality parameters by employing multilayer perceptron (MLP) neural network and the radial basis function (RBF) neural network, using data collected from three district municipalities. Two input combination models, MLP-4-5-4 and MLP-4-9-4, were trained, verified, and tested for their predictive performance ability, and their physicochemical prediction accuracy was compared by using each model’s observed data with the predicted data. The MLP-4-5-4 model showed a better understanding of the data sets and water quality predictive ability giving an MSE of 39.06589 and a correlation coefficient (R^2^) of the observed and the predicted water quality of 0.989383 compared to the MLP-4-9-4 model (R^2^ = 0.993532, MSE = 39.03087). These results apply to natural water resources management in South Africa and similar catchment systems. The MLP-4-5-4 system can be scaled up for future water quality prediction of the Waste Water Treatment Plants (WWTPs), groundwater, and surface water while raising awareness among the public and industry on future water quality.

## 1. Introduction

Water quality plays an essential role in any aquatic system, such as reflecting the degree of water pollution [1] and influencing the growth of aquatic organisms [2]. Predicting future water quality changes is a prerequisite for early water pollution control [3] and plays a crucial role in environmental monitoring, ecosystem management, and human health [1]. As a result, water quality prediction has tremendous practical significance [4,5,6] as an essential means of preventing water pollution in any catchment [7]. As influenced by natural and human-induced occurrences [8], the water quality of any catchment serves as scientific evidence for economic development, commercial planning, and water resources protection from future contamination of that catchment [8]. Therefore, water quality monitoring and prediction are of utmost importance to public health and are mandatory and crucial for better managing accessible water resources and building up various remediation strategies [9].

Traditionally, water quality evaluation and monitoring tools, such as the Water Quality Index (WQI), have been considerably used by researchers worldwide to evaluate the surface water quality because of its capability to summarize several water quality parameters into one numeric value, along with a defined scale of water quality [10,11,12,13,14,15,16]. Despite its widespread use, there is still a limitation on the index system as much of the data used cannot correlate with an index [17] and therefore insufficient to predict future water quality changes. The water pollution process is so complex that it is not only affected by natural factors but also anthropogenic factors such as social and economic development, resulting in a water environment system with strong nonlinear and non-deterministic characteristics [18]. Therefore, the traditional linear prediction model cannot fully reflect its changing regulation and cannot accurately predict its water quality. Owing to an increase in data scale and the growing need to investigate ways and means of linking together land use, pollutant loading and disposal, water quality, and ecosystem impacts, mathematical techniques and models that can efficiently model and predict water quality have been developed [19]. These modeling techniques can systematically and methodically understand the cause-and-effect relationships and assess water quality changes [20]. This ability is crucial to forecasting the variation trend of water quality at a particular time in the future [21].

In recent decades, the non-mechanism model has become a hotspot for research on water quality prediction modeling. As such, various water quality prediction techniques such as Autoregression (AR) [22,23], Moving Average (MA) [4,24], Exponential Smoothing (ES) [25], Hybrid Methods (HM) [26,27,28], multiple linear regression (MLR) [18], and the Autoregressive Integrated Moving Average (ARIMA) [29] have been used to predict and forecast the dependent variable in a time series [4,22,23,24,25,26,27,28,29]. The characteristic of these methods is establishing a water quality prediction model with a specific algorithm from the perspective of the variation in water quality data and without considering the relationship of the water pollution and the changing mechanism. Among these techniques, the multilayer perception (MLP) model has mostly outperformed others in precision and accuracy [27] and is the most widely used architecture [30]. MPL is a feed-forward ANN model that maps sets of input data into appropriate outputs. It uses a supervised learning technique that involves the use of the backpropagation algorithm [31]. This is probably the reason behind the increasing popularity of ANNs in the field of water quality prediction [6,7,8,21,30,32,33,34,35,36,37,38,39,40] and environmental analysis [27,41,42,43,44,45,46,47], that is, researchers can utilize ANN to model nonlinear and complex phenomena even if they do not fully understand the underlying changing mechanisms [48,49], hence the increased use of ANNs in water quality classification and prediction [50].

In South Africa, as would be in any developing country, economic development has led to the gradual degradation of the nation’s water resources system [51], but the extent and rate of water quality decline [52,53,54] have not been consistently and systematically measured. A few studies have utilized ANN to model and predict stream flow [55], mine water quality [56], and water demand [57] in South Africa, but none have focused on water quality prediction in a water environment system with more than one source of pollutants. In cognizance of a need to expand the modeling and prediction of water quality in South Africa, motivated by the successful applications in modeling non-linear system behaviors in a wide range of areas, ANNs are used to predict water quality parameters study. There are no current studies on modeling and predicting the physicochemical properties of water quality for natural water resources that have been conducted in South Africa. Therefore, this study uses standard water quality measuring techniques to analyze eight physicochemical parameters and further employs two input combination models (MLP-4-5-4 and MLP-4-9-4) with a multilayer perceptron feed-forward ANN to test their predictive performance required to reach input combinations capable of forecasting, with accuracy, the physicochemical parameters of selected rivers and WWTPs of three district municipalities, Eastern Cape, South Africa. This paper’s findings can be used as a baseline study for future water quality prediction in South Africa. The built networks can be scaled up and may be used to predict future water quality for any other area with the parameters studied in this work, locally and internationally. The objectives of this study are to (1) obtain the best model fits to predict water quality parameters by employing multilayer perceptron (MLP) neural network, and the radial basis function (RBF) neural network, using data collected from three district municipalities; (2) evaluate the performance of each modeling approach using observed data versus predicted data from each model; and (3) compare the performance of these two modeling approaches in terms of prediction accuracy.

The rest of this paper is organized as follows: Section 2 provides the theoretical foundations of ANNs, i.e., a glance at the application of ANNs in water quality research and prediction. Section 3 describes the study’s materials and methods, i.e., the study area, study data, and the principles of the ANN network. Section 4 presents Tests and Results, i.e., applying the results, with corresponding analyses and discussions, and the experimental conclusions drawn from the two MLPs models employed in this study. Finally, the conclusions and future work are discussed in Section 5.

## 2. Theoretical Foundations and Application in Water Quality Prediction

### 2.1. Principles of ANN

**Figure 1:**
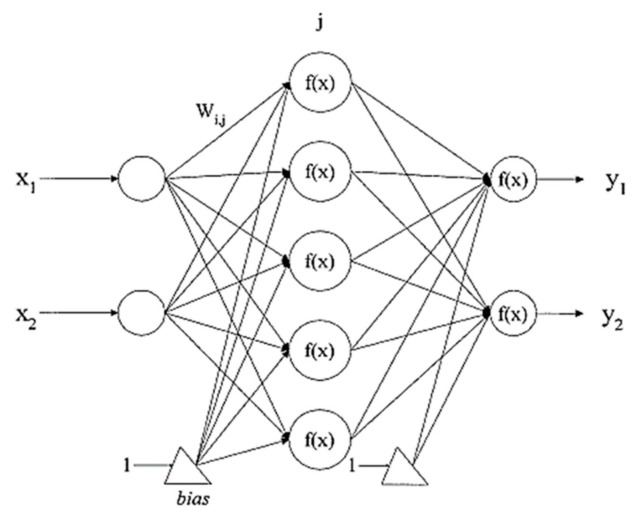
ANN with the interconnecting lines representing the weights associated with interconnections between the neurons (Adapted from [60]).

An artificial neural network (ANN) is a computing system animated by studies of the brain and nervous system [37] as in the human brain [18]. ANN carries out perfect mathematical complex systems and is based on a system of interconnected “neurons” [36,48,58] forming the basis of neural network operation. The network has computational models that are defined by four parameters: (i) processing elements known as neurons, (ii) a topology comprising weighted connections between neurons, (iii) a learning algorithm for training the network, (iv) a recall algorithm for testing or classifying purposes [59]. The neurons are interconnected according to a particular architecture/topology/structure to achieve pattern recognition in data [59]. The most widely used architecture is Multilayer Perceptron (MLPs), with only three layers in many types of feed-forward ANNs shown in Figure 1. The interconnecting links have a numeric weight updated during the learning process and allow long-term storage in the network [60]. The structure of neural networks has three layers: input neurons that receive data from an external source, hidden neurons with input and output signals that remain in the network, and output neurons that send data to an external source. The layers consist of summing units, activation functions, bias b, weight matrix W, and output vector. Each component of the input X is connected to each neuron through weight matrix W. Each neuron has an activation function f, bias b, and an output Y (Figure 1) [60].

In recent years, Artificial Intelligence (AI) earned enormous advances in various uses including solving complex and non-linear challenges [16,27,36,56,57,61]. Additionally, AI is regarded as a generally complementary method to conventional procedures or complete systems that can be used to execute modeling, forecasting, and optimization at full speed [62]. AI high technologies relate to the artificial neural network, genetic algorithm, and expert system chemometric techniques. The utilization of ANN in water engineering and environmental sciences has been pointed in many studies [33,63,64] due to its ability to show the hidden relationship in historical records, making it easy to predict and forecast water quality.

### 2.2. ANN’s Application to Water Quality Prediction

ANN models have several advantages over traditional physical and statistical models. The data needed for ANN models can be collected relatively easily. Moreover, the models are less sensitive to data insufficiency; the structures are flexible, non-linear, and robust, and can handle vast amounts of data and data at different scales [60,61,62,63,64,65]. As a result, many researchers have explored artificial neural networks (ANN), multilayer perceptron (MLP) and feed-forward neural networks (FFNN) [40,66,67,68], to predict; forecast, and model future water quality in groundwater [2,48], surface water [1,6,7,9,31,33,40,42,58,67,69,70,71,72,73,74,75], and wastewater treatment plants [68,76]. A review on water quality prediction by [58] for a period 2008–2019 concluded that the MLP architecture in ANN was the widely used architecture to complete prediction tasks during this period. The common reason behind the MLP outperforming other architectures, across all literature, lies in the MLP’s ability to approximate any relationship between input(s) and output(s) through the typical three layers and its advantage of being easy to use [1,2,6,7,9,31,33,40,42,48,58,59,60,61,62,63,64,65,66,67,68,69,70,71,72,73,74,75,76]. Therefore, the MLP architecture in ANN is a tested and suitable method for water prediction in a complex system like a river system.

## 3. Materials and Methods

### 3.1. Sample Collection

**Figure 2:**
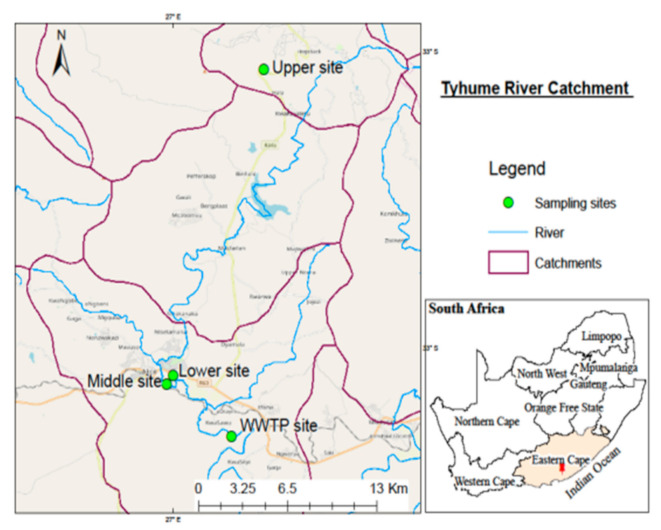
The map of the Tyhume River Catchment demonstrating the study region with sampling sites.

**Figure 3:**
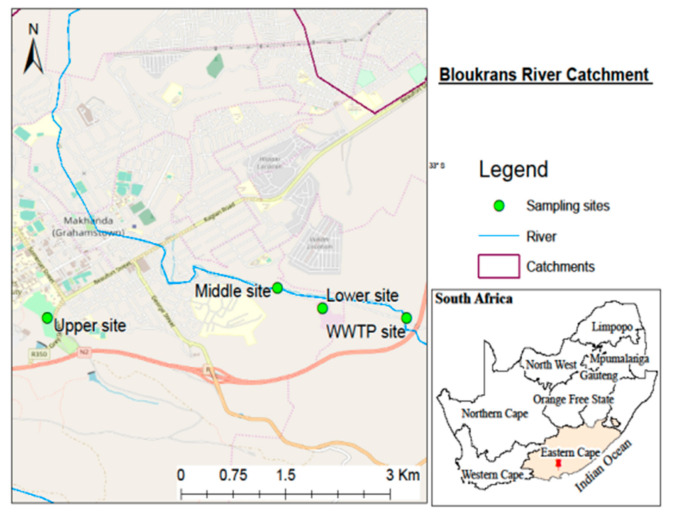
The map of the Bloukrans River Catchment demonstrating the study region with sampling sites.

**Figure 4:**
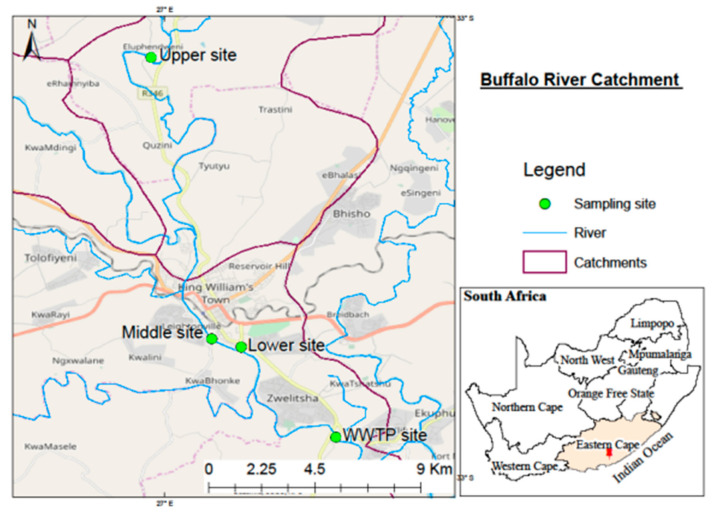
The map of the Buffalo River Catchment demonstrating the study region with sampling sites.

Water samples were collected from the Tyhume River (Raymond Mhlaba Municipality), Bloukrans River (Makhanda Municipality), Buffalo River (Buffalo City Metropolitan), and WWTPs found on the banks of these rivers in the Eastern Cape Province of South Africa. Figure 2, Figure 3 and Figure 4 show the maps and sampling sites for the respective catchments. Samples were collected from four key sampling sites (Upper, Middle, Lower, and WWTPs) in each of the three municipalities.

For each river, samples were collected from three sites: the upper stream, middle stream, lower stream, and wastewater treatment plants (influent and effluent). Samples were brought to the laboratory in a cooler box containing ice packs to preserve the temperature. The analysis of samples was performed within 48 h from the time of sampling. Table 1 represents the geographic database concerning the selected rivers’ sampling points, district municipalities, and the complete site description.

### 3.2. Physicochemical Analysis

Water samples were analyzed for physicochemical parameters by utilizing standard methods. Eight (8) physicochemical parameters were measured from fifteen sampling sites. The pH, Electrical Conductivity (EC) (uS/cm), chloride (Cl) (mg/L), Dissolved Oxygen (DO) (mg/L), and temperature (°C) were measured in situ using a portable and recalibrated HANNA Multi-parameter HI 9829 (Hanna Instruments Inc, Kallang, Singapore) meter according to the manufacturer’s guidelines. Turbidity was measured using the Turbidimeter TN-100 (Merck KGaA, Darmstadt, Germany) using the manufacturer’s guidelines. Sulphate (SO_4_^2−^) (mg/L) and phosphate (PO_4_^3−^) (mg/L) (mg/L were analyzed using photometer AL410 (Aqualytic, Dortmund, Germany) according to the manufacturer’s guidelines.

### 3.3. Artificial Neural Network (ANN)

Two types of feed-forward ANN, namely, multilayer perception (MLP) and radial basis function, were evaluated using Statistica version 13.2 software (Round Rock, Texas, USA). The artificial neural network was trained by employing the MLP with a hidden layer of 3 to 10 neurons, the Broyden–Fletcher–Goldfarb–Shanno training algorithm, and a network approximation error of 1 × 10^−14^. In this work, a feed-forward backpropagation (BP) is adopted in an artificial neural network to determine a gradient needed in the computation of the weights for the network, which is then used to construct classifiers for water quality prediction in the study areas. Each neuron in the network computes a weighted sum of its input signals to generate an internal activity level $a_{i}$,

(1) $$a_{i}=\Sigma_{J=1}^{n}\;w_{iJ}x_{ij}-w_{i^{0}}\;$$

where *x_ij_* is the *j*th input to the *i*th neuron, *w_ij_* is the weight associated with the *j*th input, and *w_i_*_0_ is the threshold associated with neuron *i*. The internal activity is passed via a nonlinear activation function $\beta_{i}$ to generate the output of the neuron $\gamma_{i}$,

(2) $$\gamma_{i}=\beta_{i}\left(\alpha_{i}\right)$$

After each *y_i_* is obtained, an activation function is used to adjust it. The standard sigmoid function is of the form,

(3) $$B_{i}\left(a_{i}\right)=\frac{1}{1+\exp\left(-a_{i}\right)}$$

The output of the activation functions *β_i_* for the neurons becomes the input for the neurons at downstream layers. The eventual output of the model is a result of the *β_i_* at the output layer. The error of hidden layers is minimized by propagating back the error desired for the output layer. The weights of the connection $\omega_{ij}$ are optimized according to the generalized Delta Rule during the training process to reach the neural networks’ desired input and output relationship. The error function, minimized by the backpropagation algorithm, is the average sum of the squares of the errors for all the outputs, and it is defined as follows,

(4) $$\delta =\sum_{i}{\left(\gamma_{i}-O_{i}\right)}^{2}$$

A simplified learning procedure for ANNs is summarized as follows: (1) supply the neuron network with training data including input variables and desired target outputs; (2) attain how closely the neuron networks outputs mates the target outputs; (3) optimize the weights of the connection between the neurons, so the neuron network yields better approximations of the target outputs; (4) keep on adjusting the weights until a specific desired accuracy is attained.

#### 3.3.1. Optimal selection of ANN model

The optimal architecture of the network was set and kept constant according to the empirical formula

(5) $$M=\sqrt{i+0}+c$$

where *M* represents the number of hidden layer nodes, *i* is the number of input sets, zero is the number of output sets, and *c* is a constant number ranging from 0 to 10.

#### 3.3.2. Selection of Input and Output Variables

Specific parameters were chosen from the ten initial settings by factorial analysis that demonstrated that the water quality was primarily affected by specific physicochemical properties. The physicochemical parameters are chloride, sulfate, temperature, phosphate, pH, electrical conductivity, turbidity, and dissolved oxygen. The significant stage of developing the ANN model is to decide the model input variables, which have a considerable influence on the performance model. The input layers (dependent variables) were set with four neurons: temperature, chloride, sulfate, and phosphate, whereas the output layers (independent variables) have four neurons: pH, electrical conductivity, turbidity, and dissolved.

#### 3.3.3. Data Preprocessing and Evaluation of the ANN Model’s Performance

Before the network is presented with the input data, a normalization procedure is required since mixing variables with large and small magnitudes confuses the learning algorithm on each variable’s importance, resulting in the rejection of variables with a smaller magnitude. Normalization scales the minimum value to 0 and the maximum value to 1. The coefficient of correlation (*R*^2^), mean square error (MSE), and root mean square error (RMSE) were employed to evaluate the model’s performance. The general formula of $R^{2}$, MSE, and RMSE are mathematically indicated in Equations (6)–(8) as follows:

(6) $$R^{2}=1-\frac{\sum {\left(x_{i}-y_{i}\right)}^{2}}{\sum y_{i}{}^{2}-\frac{\sum y_{i}{}^{2}}{n}}$$

(7) $$MSE=\frac{1}{n}\overset{n}{\underset{i=1}{\sum }}{\left(y_{i}-{\hat{y}}_{0}\right)}^{2}$$

(8) $$RMSE=\sqrt{\frac{1}{n}\overset{n}{\underset{i=1}{\sum }}{\left(y_{i}-{\hat{y}}_{0}\right)}^{2}}$$

### 3.4. Training and Testing Network

Experimental data were categorized into training and testing sets. The training set was employed to generate the ANN model; validating and testing sets were used to confirm the model’s generalization competencies. The measured data collection is divided into 70% of the training set, 10% of validation, and testing sets.

## 4. Tests and Results

The statistical variables of annual water quality parameters for the Tyhume, Buffalo, and Bloukrans Rivers and their municipal wastewater treatment plants are given in Table 2. The data was divided into 70% of the training set, 10% of validation, and testing sets and fed into the ANN model. Table 3 gives a summary of the two input combination networks, MLP-4-5-4 and MLP-4-9-4, used in this study.

Table 3 shows the summary of two input combination networks (MLP-4-5-4 and MLP-4-9-4) with a multilayer perceptron feed-forward ANN. MLP-4-5-4 produced a correlation coefficient (R^2^) value of 0.989383 with a mean square error (MSE) value of 39.03087, and MLP-4-9-4 produced an R^2^ value of 0.993532 with an MSE value of 39.06589.

According to the test data percentage difference for the MLP 4-5-4 and MLP 4-9-4 networks (Table 4), both networks adequately understood the relationship between the data sets. The percentage difference for the first test data set was comparable/similar, and that of the second set showed a difference in predictive ability. The lowest percentage difference (3.48%) for the second test data set was given by MLP 4-5-4, and therefore, this network best understood the relationship between the independent variables and the pH of the water.

The percentage difference in the test data for electrical conductivity (Table 5) of both networks (MLP 4-5-4 and MLP 4-9-4) was within the acceptable range (10% limit). MLP 4-5-4 demonstrated a better predictive ability as it exhibited the lowest percentage difference (1.6%).

Turbidity test data set percentage differences (Table 6) for MLP 4-5-4 and MLP 4-9-4 were above the 10% limit, suggesting that both networks did not adequately understand the relationship between the investigated independent variables and turbidity. The results indicate a nonsignificant effect of the studied variables on the test variable.

The percentage difference (Table 7) for the MLP 4-5-4 and MLP 4-9-4 networks’ dissolved oxygen (DO) test data (Table 7) was within the acceptable limit of 10%. The systems understood the relationship between the data sets. MLP 4-5-4 exhibited a percentage difference significantly lower (5.42% difference) than that of MLP 4-9-4 for the first data set, while MLP 4-9-4 exhibited the slightest percentage difference for the second test data, 1.60% different from MLP 4-5-4. These results suggest that the best predictive ability was demonstrated by MLP 4-5-4.

## 5. Discussions

This study uses standard water quality measuring techniques to analyze eight physicochemical parameters in water samples collected from Wastewater Treatment Plants (WWTPs) and three major rivers. Furthermore, the study employs two input combination models (MLP-4-5-4 and MLP-4-9-4) with a multilayer perceptron feed-forward ANN to test their predictive performance required to reach input combinations capable of forecasting water quality accurately. The results obtained from the test data percentage differences of the two networks show that both networks adequately understood the relationship between the training and testing data sets, with the MLP 4-5-4 model showing better generalization competencies in understanding the relationship between the independent variables and the investigated physicochemical parameters of the water samples (Table 4, Table 5 and Table 7). However, the results showed a nonsignificant effect between the studied variables and the turbidity test (Table 6) as the experimental and predicted percentage difference values for both networks are above the 10% limit.

The observed percentage difference (above the 10% limit) in experimental, and predicted test results between the turbidity and the independent variables can be interpreted several ways. It may be that there truly is no significant effect of the studied variables on water turbidity, suggesting that the built systems did not adequately understand the relationship between the investigated independent variables and turbidity. Alternatively, it could be that there is a significant effect, but the MLP-4-5-4 and MLP-4-9-4 models’ predictive ability in the present study was not sensitive enough to test data due to a variety of potential factors. First, this result reflects on the effect of other parameters on the water turbidity. That is, the studied variables may not have a significant effect on the test variable. More variables would have to be considered in future studies. Second, the ANN model depends significantly on data quantity [58]. As a result, it may not be advised to utilize comparatively small data for input variables as some valuable data may be lost in short-term data, resulting in unsatisfactory predicted results [77]. Third, the input combination could be a factor in the observed results. Data division is a crucial stage in the method of process modeling. Reaching a precise forecast exploiting an artificial neural network is determined by selecting an excellent input combination model [78]. Nevertheless, both networks’ predictability performance in other variables tests showed significant results, implying that the experimented and predicted data are strongly correlated.

The MLP-4-5-4 and MLP-4-9-4 both showed commendable predictive performance and input combinations capable of forecasting water quality and supporting this study’s objectives. The MLP-4-5-4 produced a higher correlation coefficient (R^2^) and lower MSE than the MLP-4-9-4 network (Table 3). The higher the R^2^ and lower the RSME, the better the model fits the dataset [79]. These results suggest that the MLP method was able to learn the system significantly well. This study’s outcomes are consistent with other studies conducted in South Africa [55,56,80]. A study by Isiyaka et al. 2019 [78] used a multilayer perceptron feed-forward artificial neural network to predict the level of water pollution. The authors reported the best input combination and the highest R^2^ = 0.999 value with the least RMSE = 0.159 and, based on these findings, concluded that ANN could also predict the water quality index with a high level of accuracy using less complex input variables that can be adopted for water quality prediction and modeling in the subsequent analysis [78]. These results agree with the findings of the present study (Table 3). Several other studies are consistent with the present study and conclude, based on similar findings, that the ANN model can easily classify and predict water quality with the justifiable output [19,20,50,55,81,82,83,84,85,86,87,88].

The present study is essential because the MLP-4-5-4 system can be scaled up and used for the future water quality prediction of the Waste Water Treatment Plants (WWTPs), groundwater, and surface water at the municipal, regional, and national scales. Municipalities and other water quality bodies can benefit from this research’s outcomes. More significantly, the model can help manage natural resources and raise awareness among the public and industry. Furthermore, the MLP-4-5-4 system can help reduce water quality decision-maker uncertainty using a novel and refined model to predict and classify WWTPs and river water variables’ quality with acceptable precision. Furthermore, the results can be used to manage water quality in the study area and other regions.

## 6. Conclusions and Future Studies

The ANN model was developed to test its predictive performance on the quality of river water and WWTPs and has a great opportunity as a predictive tool. Most notably, the method of MLP was able to learn the system reasonably well. The MLP 4-5-4 network showed the best predictive ability for water quality. The application of this model to the river basins in the study area has shown the possibility of using available data in a given catchment to predict water quality while recognizing the fact that such data-intensive models as ANN may not be successful in developing countries where data is inadequate, a notable limitation in the present study. Future research should direct attention to applying the same techniques to other catchments and provinces and consider relatively long data series to reasonably compare the performance of the models in water resources.

Furthermore, we intend to focus on water quality prediction in extreme weather conditions and the building of a uniform model for multiple catchments at a one-time step. This is crucial to testing the effect of spatial and temporal variations on water quality modeling and prediction since water quality varies at spatial and temporal scales. This research line is crucial to understanding the means of linking together land use, pollutant loading and disposal, water quality, and ecosystem impacts to efficiently model and predict water quality. Therefore, the ANN model is a golden and valid instrument that optimizes the observational network by determining important monitoring sites and predicting river water variables’ quality with acceptable precision. However, while the results derived from ANN in this study are not necessarily statistically significantly better than the results derived from a combination of descriptive statistics, the water environment system is a very complex system with nonlinear solid and non-deterministic characteristics. As such, these results offer more accurate and comprehensive water prediction data. To improve prediction accuracy, accommodating uncertainty associated with the water environment system, modern algorithms are suitable for time-sequential prediction, such as the ensemble approach, transfer learning technology, and evidence theory can be used.

## Figures and Tables

**Table 1:** Descriptive sampling sites and coordinates for rivers and wastewater treatment plants (WWTPs).

River	Site	Full Site Description	GPS Coordinate	
			Latitude	Longitude
**Makhanda Municipality**				
**Bloukrans**	GU	Upper site of the Bloukrans River	33.31774167	26.52194444
	GM	Middle site of the Bloukrans River	33.31427500	26.55166667
	GL	Lower site of the Bloukrans River	33.31780556	26.56833333
	GE	Influent site of WWTP of Grahamstown region	33.31667500	26.55750000
	GI	Effluent site of WWTP of Grahamstown region		
**Buffalo City Metropolitan**				
**Buffalo**	BU	Upper site of the Buffalo River	32.78991389	27.36916667
	BM	Middle site of the Buffalo River	32.89703333	27.39277778
	BL	Lower site of the Buffalo River	32.93447500	27.44027778
	BE	Influent site of WWTP of King William’s Town region	32.89969722	27.40305556
	BI	Effluent site of WWTP of King William’s Town region		
**Raymond Mhlaba Municipality**				
**Tyhume**	AU	Upper site of the Tyhume River	32.61067778	26.90944444
	AM	Middle site of the Tyhume River	32.79636667	26.84583333
	AL	Lower site of the Tyhume River	32.82713889	26.88833333
	AE	Influent site of WWTP of Alice region	32.79108611	26.85000000
	AI	Effluent site of WWTP of Alice region		

U—Upper stream, M—middle stream, L—lower stream, E-WWTPs effluent, I—WWTPs influent; G-Bloukrans River, GI/GE—Makhanda WWTP B—Buffalo River, BI/BE—King William’s Town WWTP; A—Tyhume River, AI/AE—Alice WWTP.

**Table 2:** Statistical variables of annual water quality parameters of the river basins of Tyhume, Buffalo, and Bloukrans Rivers and their municipal wastewater treatment plants.

Sample Area	Temperature (°C)	Chloride (Cl) (mg/L)	Sulphate (SO_4_^2-^) (mg/L)	Phosphate (PO_4_^3-^) (mg/L)	pH	Turbidity (NTU)	Electrical Conductivity (EC) (mS/m)	Dissolved Oxygen (DO) (mg/L)
**AU**	12.77	4.00	4.00	0.06	8.08	7.32	11.10	7.43
**AM**	15.66	7.67	4.00	0.42	7.05	18.21	26.02	7.57
**AL**	14.88	4.00	4.00	0.04	9.43	12.36	40.29	7.58
**AE**	18.38	28.00	65.34	0.04	7.16	6.57	64.89	7.22
**AI**	19.32	4.00	48.44	0.04	7.29	15.28	72.82	4.85
**BU**	17.24	180.67	9.67	0.04	7.46	18.17	50.35	7.26
**BM**	18.22	4.00	52.50	0.30	7.71	23.27	61.74	7.02
**BL**	17.95	4.00	119.00	0.04	7.89	14.34	75.33	7.11
**BE**	19.25	4.00	64.22	0.28	7.27	23.44	85.24	6.59
**BI**	19.50	16.00	32.84	0.13	7.27	191.00	102.88	4.77
**GU**	13.24	4.00	45.84	0.04	6.22	9.14	73.42	7.25
**GM**	15.63	69.67	127.33	0.04	7.28	30.96	230.60	6.03
**GL**	14.75	83.33	156.50	0.55	6.41	89.82	223.50	6.27
**GE**	17.93	9.67	136.78	0.35	7.16	137.00	209.54	5.84
**GI**	21.51	4.00	63.33	0.43	7.52	206.15	226.27	4.72

U—Upper stream, M—middle stream, L—lower stream, E—WWTPs effluent, I—WWTPs influent; G—Bloukrans River, GI/GE—Makhanda WWTP B—Buffalo River, BI/BE—King William’s Town WWTP; A—Tyhume River, AI/AE—Alice WWTP.

**Table 3:** Summary of MLP-4-5-4 and MLP-4-9-4 active networks.

Network Name	R^2^	MSE	Training Algorithm	Error Function	Hidden Activation	Output Activation
**MLP 4-5-4**	0.989383	39.03087	BFGS 88	SOS	Logistic	Logistic
**MLP 4-9-4**	0.993532	39.06589	BFGS 130	SOS	Tanh	Exponential

**Table 4:** Experimental and predicted values for pH generated by MLP 4-5-4 and MLP 4-9-4 networks. Sample: Training, Test, Validation.

Sample Area	Sample	Experimental pH Values	Predicted pH Values (MLP 4-5-4)	% Difference	Predicted pH Values (MLP 4-9-4)	% Difference
**AU**	Training	8.080000	8.071202	0.11	8.086230	0.08
**AM**	Training	7.050000	7.048911	0.02	7.097960	0.68
**AL**	Training	9.430000	9.330070	1.06	9.444249	0.15
**AE**	Test	7.160000	7.441819	3.94	7.393835	3.27
**AI**	Training	7.290000	7.442833	2.10	7.351340	0.84
**BU**	Training	7.460000	7.399046	0.82	7.466864	0.09
**BM**	Test	7.710000	7.441837	3.48	7.387560	4.18
**BL**	Training	7.890000	7.441830	5.68	7.621972	3.40
**BE**	Training	7.270000	7.442062	2.37	7.475793	2.83
**BI**	Training	6.270000	7.443619	18.72	7.085941	13.01
**GU**	Training	6.220000	6.220000	0.00	6.221081	0.02
**GM**	Validation	7.280000	7.426453	2.01	7.733750	6.23
**GL**	Training	6.410000	6.552220	2.22	6.384595	0.40
**GE**	Validation	7.160000	7.441715	3.93	7.994136	11.65
**GI**	Training	7.520000	7.448825	0.95	7.622527	1.36

U—Upper stream, M—middle stream, L—lower stream, E-WWTPs effluent, I—WWTPs influent; G—Bloukrans River, GI/GE—Makhanda WWTP B—Buffalo River, BI/BE—King William’s Town WWTP; A—Tyhume River, AI/AE—Alice WWTP.

**Table 5:** Experimental and predicted values for pH generated by MLP 4-5-4 and MLP 4-9-4 networks. Sample: Training, Test, Validation.

Sample Area	Sample	Experimental EC	Predicted EC MLP 4-5-4	% Difference	Predicted EC MLP 4-9-4	% Difference
**AU**	Training	11.1000	12.7558	14.92	11.1286	0.26
**AM**	Training	26.0200	27.4272	5.41	41.8489	60.83
**AL**	Training	40.2900	11.9350	70.38	15.6602	61.13
**AE**	Test	64.8900	62.7317	3.33	61.9240	4.57
**AI**	Training	72.8200	86.7355	19.11	75.6735	3.92
**BU**	Training	50.3500	60.1378	19.44	51.4812	2.25
**BM**	Test	61.7400	62.7808	1.69	59.0714	4.32
**BL**	Training	75.3300	62.7223	16.74	81.7645	8.54
**BE**	Training	85.2400	67.6846	20.60	66.1918	22.35
**BI**	Training	102.8800	108.4363	5.40	101.2073	1.63
**GU**	Training	73.4200	73.9280	0.69	73.0470	0.51
**GM**	Validation	230.6000	62.9033	72.72	93.0826	59.63
**GL**	Training	223.5000	226.2581	1.23	223.6889	0.08
**GE**	Validation	209.5400	62.7630	70.05	119.5078	42.97
**GI**	Training	226.2700	214.8109	5.06	226.2055	0.03

U—Upper stream, M—middle stream, L—lower stream, E-WWTPs effluent, I—WWTPs influent; G—Bloukrans River, GI/GE—Makhanda WWTP B—Buffalo River, BI/BE—King William’s Town WWTP; A—Tyhume River, AI/AE—Alice WWTP.

**Table 6:** Experimental and predicted values for turbidity generated by MLP 4-5-4 and MLP 4-9-4 networks. Sample: Training, Test, Validation.

Sample Area	Sample	Experimental Turbidity	Predicted Turbidity MLP 4-5-4	% Difference	Predicted Turbidity MLP 4-9-4	%Difference
**AU**	Training	7.3200	7.3200	0.00	7.4476	1.74
**AM**	Training	18.2100	7.5026	58.80	10.3960	42.91
**AL**	Training	12.3600	7.3200	40.78	8.0410	34.94
**AE**	Test	6.5700	17.3447	164.00	16.5765	152.31
**AI**	Training	151.200	147.5284	2.43	150.3962	0.53
**BU**	Training	18.1700	15.3185	15.69	13.9046	23.47
**BM**	Test	23.2700	17.4291	25.10	16.9588	27.12
**BL**	Training	14.3400	17.3287	20.84	25.6735	79.03
**BE**	Training	23.4400	29.5244	25.96	21.8990	6.57
**BI**	Training	191.0000	201.9652	5.74	192.3942	0.73
**GU**	Training	9.1400	7.3200	19.91	8.2733	9.48
**GM**	Validation	3.9600	17.2437	335.45	32.7986	728.25
**GL**	Training	89.8200	87.0369	3.10	89.3686	0.50
**GE**	Validation	137.5000	17.3304	87.40	55.9932	59.28
**GI**	Training	206.1500	206.1500	0.00	205.9516	0.10

U—Upper stream, M—middle stream, L—lower stream, E-WWTPs effluent, I—WWTPs influent; G—Bloukrans River, GI/GE—Makhanda WWTP B—Buffalo River, BI/BE—King William’s Town WWTP; A—Tyhume River, AI/AE—Alice WWTP.

**Table 7:** Experimental and predicted values for dissolved oxygen generated by MLP 4-5-4 and MLP 4-9-4 networks. Sample: Train, Test, Validation.

Sample Area	Sample	Experimental DO	Predicted DO MLP 4-5-4	% Difference	Predicted DO MLP 4-9-4	% Difference
**AU**	Training	7.430000	7.580000	2.02	7.420167	0.13
**AM**	Training	7.570000	7.570000	0.00	7.602289	0.43
**AL**	Training	7.580000	7.580000	0.00	7.596899	0.22
**AE**	Test	7.220000	7.137646	1.14	6.746576	6.56
**AI**	Training	4.850000	4.907170	1.18	4.722991	2.62
**BU**	Training	7.260000	7.261512	0.02	7.239937	0.28
**BM**	Test	7.020000	7.133850	1.62	7.021295	0.02
**BL**	Training	7.110000	7.138362	0.40	7.022525	1.23
**BE**	Training	6.590000	6.638667	0.74	6.598599	0.13
**BI**	Training	4.770000	4.726695	0.91	4.721815	1.01
**GU**	Training	7.250000	7.580000	4.55	7.251133	0.02
**GM**	Validation	6.030000	7.142718	18.45	6.927154	14.88
**GL**	Training	6.270000	6.214789	0.88	6.331988	0.99
**GE**	Validation	5.840000	7.138329	22.23	6.721605	15.10
**GI**	Training	4.720000	4.720000	0.00	4.769756	1.05

U—Upper stream, M—middle stream, L—lower stream, E-WWTPs effluent, I—WWTPs influent; G—Bloukrans River, GI/GE—Makhanda WWTP B—Buffalo River, BI/BE—King William’s Town WWTP; A—Tyhume River, AI/AE—Alice WWTP.

## Data Availability

Samples are not available from authors.
